# Whole-genome sequencing targets drug-resistant bacterial infections

**DOI:** 10.1186/s40246-015-0037-z

**Published:** 2015-08-05

**Authors:** N. V. Punina, N. M. Makridakis, M. A. Remnev, A. F. Topunov

**Affiliations:** Bach Institute of Biochemistry, Russian Academy of Science, Moscow, 119071 Russia; Tulane University School of Public Health and Tropical Medicine, New Orleans, LA 70112 USA; The Federal State Unitary Enterprise All-Russia Research Institute of Automatics, Moscow, 127055 Russia

## Abstract

During the past two decades, the technological progress of whole-genome sequencing (WGS) had changed the fields of Environmental Microbiology and Biotechnology, and, currently, is changing the underlying principles, approaches, and fundamentals of Public Health, Epidemiology, Health Economics, and national productivity. Today’s WGS technologies are able to compete with conventional techniques in cost, speed, accuracy, and resolution for day-to-day control of infectious diseases and outbreaks in clinical laboratories and in long-term epidemiological investigations. WGS gives rise to an exciting future direction for personalized Genomic Epidemiology. One of the most vital and growing public health problems is the emerging and re-emerging of multidrug-resistant (MDR) bacterial infections in the communities and healthcare settings, reinforced by a decline in antimicrobial drug discovery. In recent years, retrospective analysis provided by WGS has had a great impact on the identification and tracking of MDR microorganisms in hospitals and communities. The obtained genomic data are also important for developing novel easy-to-use diagnostic assays for clinics, as well as for antibiotic and therapeutic development at both the personal and population levels. At present, this technology has been successfully applied as an addendum to the real-time diagnostic methods currently used in clinical laboratories. However, the significance of WGS for public health may increase if: (a) unified and user-friendly bioinformatics toolsets for easy data interpretation and management are established, and (b) standards for data validation and verification are developed. Herein, we review the current and future impact of this technology on diagnosis, prevention, treatment, and control of MDR infectious bacteria in clinics and on the global scale.

## Introduction

Human genomics is inseparably linked to the genomics of bacteria. Bacteria share a long history with humans and play a major role in our life [152, 200]. Beneficial utilization of bacterial products can provide key solutions to many pressing problems on the planet, from environmental pollution to human diseases. Investigation of bacterial pathogens remains agenda priority mainly due to two additional reasons: (i) over 13 % of the world’s deaths are related to bacterial infectious disease (including respiratory diseases and tuberculosis (TB)) every year [79, 250], and (ii) the growth of ancient pathogen re-emergence is driven by steadily increasing resistance to multiple widely used antimicrobial agents [59, 60, 249]. Despite the importance and utility of bacteria, until quite recently, little was known about their genomic structure.

During the last two decades, bacteria genomics is rapidly changing, mostly through the evolution of whole-genome sequencing (WGS) technologies. Recent technical advantages significantly reduced the cost of WGS and improved its power and resolution. Since WGS tools (both chemistry and bioinformatics-wise) are changing rapidly, we will not dwell in the details of individual technologies and equipment. The variety and applicability of the major high-throughput sequencing platforms are well presented in several reviews (e.g., [149, 199, 263]).

The advent and ever-growing use of the novel WGS technologies resulted in a rapid intensification in the scope and speed of the completion of bacterial genome sequencing projects. This explosion in bacterial genomics has greatly expanded our view of the genetic and physiological diversity of bacteria. To date, over 39,000 genome projects have been started, approximately 3,000 microbes’ whole-genome sequences were completed and published [134, 181, 229], and more than 500 new species are being described every year [68, 112]. However, most of these projects were driven by the potential practical applications of the investigated microorganisms and thus missed most of the microbial diversity on the planet [133, 134, 180].

Although researchers have only scratched the surface of microbial biodiversity, the information gained has already resulted in the discovery of large numbers of pathogenic bacteria in humans. WGS technologies granted access to potential virulence determinants, disruptive targets, candidate drug compounds [85], mechanisms of pathogenicity, drug resistance and spread [62], and their evolution in pathogens. In addition, WGS analysis provided information about uncultured or difficult-to-grow bacterial strains isolated from clinical specimens [15]. Knowledge of the enormous range of microbial capacities and functional activity can address many epidemiological questions and will have broad and far-reaching implications for personalized and public healthcare in the future. In this field, potential applications of WGS can be essential for:

i. Detection, identification, and characterization of infectious microorganisms
ii. Design of novel diagnostic assays for laboratory use
iii. Assessment of multidrug resistance (MDR) or virulence repertoires in pathogens, as well candidate antimicrobial compounds in beneficial microorganisms
iv. Monitoring the emergence and spread of bacterial infectious agents in different healthcare settings [46, 69, 126]

The WGS technology is very likely to become an alternative to the traditional methods of fighting DR bacteria. Even today, this technology is already used globally as an addendum to complement conventional laboratory approaches (microscopy, pathogenic tests, mass spectrometry, conventional molecular diagnostics, techniques for vaccine and antibiotic design) in routine clinical workflow and scientific investigations [93, 96, 149]. In the future, WGS may simplify the diagnostic laboratory workflow and sample trace, as well as reduce the number and type of collected biological specimens [11, 46, 126, 138, 201]. Deploying WGS into individual genome sequencing (IGS) technology has great potential to become a part of routine personalized clinical practice (e.g., TruGenome Clinical Sequencing tests™ by Illumina Clinical Services Laboratory; Complete Genomics Platform™ by Complete Genomics BGI, Helicos Helicope™ by SeqLL; Personal Genome Project) [92]. It is further expected that WGS will permit a deep understanding of infection mechanisms, allow for more rational preventive measures [24], and reduce the risk of unnecessary infection-control interventions [228].

The growing incidence of bacterial resistance to a broad range of antibacterial drugs in hospitals and communities is a major public health threat today and a compelling reason for WGS application. MDR pathogens complicate efforts of infection control and result in considerable morbidity and mortality around the world [111, 131, 217]. Today, MDR infections are recognized as multidimensional global challenge by many health organizations [26, 232, 251]. This complex problem requires comprehensive measures to be solved [42]. It was postulated that effective problem-solving strategies should include: (i) revealing and monitoring infectious agents, (ii) tracking antibiotic resistance, (iii) developing new antimicrobial drugs, (iv) providing rational antimicrobial stewardship program in healthcare institutions in order to avoid inappropriate or excessive antibiotic use, and (v) developing unified toolsets and standards for effective worldwide data management [42, 221, 224].

Taking into account the growing concern about emerging infections, in this review, we detail the main uses and hurdles of WGS technologies in clinical practice and public health with regard to MDR bacterial infections.

### Main directions of WGS applications in MDR bacterial infections (review scope)

There are numerous possible applications of WGS in dealing with infectious disease of MDR bacteria. WGS can be used as a primary tool for:

i. Detection of multidrug susceptibility
ii. Monitoring MDR evolution and transmission dynamics of MDR pathogen
iii. Diagnosis and control of MDR infections locally and regionally
iv. Development of new tests and assays for accurate and rapid MDR bacterial diagnostics in clinics and points-of-care
v. Discovery of novel antibacterial drugs and therapeutics and assessment of their preventability

Each of these tasks is important for clinical and public health and requires methods with different levels of typing resolution. Theoretically, this problem can be addressed by reliable, quick, and low-cost WGS technology in the near future.

#### Detection of MD susceptibility

Recently introduced into routine clinical microbiological analysis, WGS has had a great impact on the study of the spectrum of genetic factors involved in MDR to microorganisms and, consequently, on the cost-effectiveness of subsequent disease treatment [214]. Rapid and accurate identification and characterization of known and new antibiotic resistance determinants and their arrangements play a key role in preventing the emergence and spread of MDR pathogenic microorganisms in any healthcare setting [214]. Current knowledge of the type of pathogen and its antibiotic resistance profile is essential for selection of therapy and development of new antibacterial drugs [106, 123, 214] and for reducing the high mortality rate in infected patients. This knowledge also has particular significance for the pathogens causing most frequent and severe types of healthcare-associated and community-acquired infections such as bloodstream (BSI), urinary tract (UTI), and wound stream infections (WSI) [170]. The MDR bacterial pathogens of international concern [36, 161, 252] are presented in Table 1.

**Table 1:** Common MDR bacterial agents of epidemiological importance causing severe infections in hospitals (H) and communities (C)

Bacterial agent	Diseases	Resistance	Example of main resistance determinants revealed in whole sequenced genomes
*Escherichia coli* (H, C)	UTI, BSI	β-Lactams (cephalosporins)	*ampC*, 2 copies of *bla*_*T*_ [74]
		Quinolones (fluoroquinolones)	*gyrA* (Ser83Leu,Asp87Asn), *parC* (Ser80Ile,Glu84Gly) [74, 188]
*Klebsiella pneumonia* (H, C)	UTI, BSI, pneumonia	β-Lactams (cephalosporins, carbapenems)	*bla*_*SHV-75*_, *bla*_*SHV-60*_, *bla*_*KPC-2*_, *bla*_*TEM-1*_, *bla*_*TEM-12*_, *bla*_*P1*_, *bla*_*CTX-M*_ [132, 145]
		Quinolones (fluoroquinolones)	*qnrA1*, *qnrB4*, *oqxAB*, *gyrA* (Ser 83Phe), *parC* (Ala339Gly, Asp641Tyr) [209]
		Amynoglycosides	*armA*, *aph* [209]
		Colistin	IS1 insertion in the *mgrB* [95, 209]
*Staphylococcus aureus* (H, C)	WSI, BSI	β-Lactams (methicillin)	*mecC* [157]
		Aminoglycosides	*aadD* [258]
		Mupirocines	*ileS-2* [258]
		Mercury resistance	*mer* operon [258]
		Antiseptic resistance	*qacA* [258]
*Streptococcus pneumonia* (C)	Pneumonia, meningitis, otitis	β-Lactams	*pbp2a*, *pbp2b*, *pbp2x*, *spr1238* [56]
		Tetracycline	*rpsJ*, *patA*, *patB* [153]
*Salmonella enterica subsp. enterica*, *Typhimurium*, *Choleraesuis* (C)	Salmonellosis, foodborne diarrhea, BS	β-Lactams	*bla*_*OXA-30*_, *ampC*, *bla*_*TEM-1*_, *bla*_*TEM-67*_ [31, 91]
		Quinolones (fluoroquinolones)	*gyrA* (Ser83Leu), *parC* (Ser80Leu), *acrAB-tolC* [31]
		Aminoglycosides	2 copies *aadA1*, 3 copies *aadA3*, *aac3*, *aph*, *strA*, *strB*, *sat-1* [91, 109]
*Shigella* spp. (C)	“Bacillary dysenteria”	β-Lactams	*bla*_*TEM-1*_, *bla*_*OXA-1*_ [257]
		Fluoroquinolones	mutated *parC* and *gyrA* [257]
		Aminoglycosides	*aadA1*, *aadA2*, *sat-1* [257]
*Neisseria gonorrhoeae* (C)	Gonorrhea	β-Lactams (3rd gen. cephalosporins)	*mtrR* (G45D, A39T), *mtrCDE* (del1327932), *penB* (G101K, A102D), *penA* (mosaic) [54, 83, 240]
		Tetracycline	*rpsJ* (V57M), *tetM* including its promoter, *penB* [54]
Coagulase-negative *Staphylococci* spp. (CoNS) (H, C)	SSI, endocarditis, and BSI	β-Lactams	*blaZ*, *mecC* [25, 173, 184]
*Enterobacter aerogenes* (H)	SSI and BSI	β-Lactams	*bla*_*TEM-24*_, 2 copies *ampC*, 3 copies *M-bla*, 4 copies *bla* [48]
		Quinolones	*gyrA* (Thr83Ile), *parC* (Ser80Ile) [47, 48]
		Aminoglycosides	*aadA1*, *aac(6′)* [47, 48]
		Rifampicin	*rpoB* (Asp252Tyr) [47, 48]
*Acinetobacter baumannii* (H, C)	BSIs, VAP, HAP, SSI, CA-UTI, ventilator-associated pneumonia	β-Lactams (3rd gen. cephalosporins)	*bla* class A, *ISAba1*, *bla*_OXA-23_, *bla*_*OXA-10*_, *bla*_*OXA-69*_, *bla*_*ampC*_, *bla*_*OXA-23*_, *bla*_*OXA-66*_, *bla*_*ADC*_ (Nigro et al. 2013 [173]); [2, 76, 83, 173, 195, 240]), *ampC*, *bla*_*OXA-51*_-like [74], *bla*_*TEM-1*_ [2], 5 copies *M-bla*, 2 copies *ampC*, *bla*_*OXA-82*_ [204]
		Amynoglycosides	Modified *armA*, *aac(3′)*, *aac(3)-la*, *aac(6ʹ)*, *aac(2ʹ)-Ib*, *aadA1*, *aadAB*, *aphA1*, *aph(3′)*, *aph6*, *strAB* [2, 71, 204, 254, 265] *adeT*, *aadA2* [204]
		Quinolones	*gyrA* (Ser83Leu), *parC* (Ser80Leu) [2, 71, 204]
	BSIs, VAP, HAP, SSI, CA-UTI, ventilator-associated pneumonia	Colistin	*pmrB* [90]
		Tetracyclines	*tetAR*, *adeB* [2, 265], *bcr* [204]
		Chloramphenicol	*cmlA*, *cmlA5*, *cat* [71], *catB6* [265], *catB8* [254], *cmr* [204]
*Pseudomonas aeruginosa* (H, C)	BSIs, VAP, HAP, SSI, CA-UTI, cystic fibrosis (CF)	β-Lactams (3rd gen. cephalosporins)	*blaI*_*MP-1*_, *oprD* [163] *ampCDR* [124]
		Quinolones	*gyrA* (Thr83Ile), *parC* (Ser87Leu) [247]
		Aminoglycosides	*aac(6′)* [163], *aph*, *ant(4′)-IIb*, *strAB* [247], *aacA29a*/*aacA29b* [124]
		Colistin	*pmrAB*, *phoPQ* [247]
		Wide range of antibacterial agents	*mexAB-oprM*, *mexXY*, *mexCD-oprJ*, *mexEF-oprN*, *mexHI-opmD*, *mexR*, *nfxB*, *mexT*, *mexG* [124]
*Mycobacterium tuberculosis* (H, C)	Tuberculosis	Rifampicin	*rpoB* (S450L) [52]
		Isoniazid	*katG* (P7 frameshift), *ptrBa*, *fadD15*, *ppsB*, *atsH* [88]
		Fluoroquinolone ethambutol amikacin para-aminosalicylic acid	*gyrB* (T500N), *embB* (D1024N), *rrs*(A514C, A1401G), *thyA* (P17L) [52]

*BSI* bloodstream infection, *SSI* surgical-site infection, *CA-UTI* catheter-associated urinary tract infection, *VAP* ventilator-associated pneumonia, *HAP* hospital-acquired pneumonia, *WSI* wound stream infection, *UTI* urinary tract infection

Many chromosome- and plasmid-mediated resistance determinants were successfully identified for most severe pathogenic bacteria using WGS technologies (Table 1). Together with data obtained by classic antimicrobial susceptibility tests [118] and genotyping methods [66], these determinants were deposited into the Antibiotic Resistance Genes Database (ARDB) [146]. Currently, there is an open catalog of more 13,000 antibiotic resistance genes, composing the resistome [253], with rich information, including resistance profile, mechanisms, requirements, epidemiology, coding sequences, and their mutations for more than 250 bacterial genera.

Revelation of the links between genetic and phenotypic traits of bacteria still remains one of the most critical issues that thwart implementation of WGS in clinical and public health practice. Determination of the genetic components of antibiotic resistance (resistant genotypes) and their correlation to resistant bacterial phenotypes can potentially promote its practical application. The possibility to ascertain the phenotypic antimicrobial resistance on the basis of genomic data has been extensively studied [196, 261]. The resistance phenotypes determined based on WGS data were compared to the results of phenotypic tests for methicillin-resistant *Staphylococcus aureus* (MRSA) [82, 103], *Clostridium difficile* [53], *Escherichia coli*, *Klebsiella pneumonia* [100, 218], and *Pseudomonas aeruginosa* [41, 124]. The analyses showed that data obtained for these bacteria through WGS can reliably predict antibiotic susceptibility phenotype, with overall sensitivity and specificity more than 95 % [53, 82, 218]. Hence, WGS may be applied as first-line antibiotic resistance screening method in clinical practice of these pathogens. However, it is important to remember that in some cases, bacterial MDR depends on the mode and level of the resistance gene expression [118]. Thus, presence of the genetic resistance determinants does not solely determine MDR phenotype and success/failure of the antibiotic therapy.

Owing to this and other facts (discussed herein), current WGS technology can be clinically applicable only as an integral part of a comprehensive state/government-approved workflow for the clinically relevant cases, e.g., typing of linezolid-resistant *Enterococcus faecium* or screening of carbapenem-resistant Enterobacteriaceae [101, 194]. Future investigations of pathogen resistance mechanisms together with establishment of robust links between genetic components and phenotypic traits in MDR bacteria will help the development of successful WGS-based antibiotic resistance tests. Development of standardized procedures for validation and verification of WGS data, as well user-friendly bioinformatics tools for quick handling and analysis of the genomic information will speed up the implementation of WGS technologies into laboratory practice. For example, one of these tools is provided by the Center for Genomic Epidemiology [136].

#### Investigation of MDR evolution and emergence dynamics

WGS has been used for the study of the evolution of resistance (or proto-resistance) to multiple drugs and its emergence in different healthcare settings [182]. Large-scale worldwide studies showed that this method could be applied to elucidate historical antibiotic resistance patterns in pathogen populations and study infection transmission mechanisms and emergence dynamics. Specifically, WGS technologies allowed uncovering the genetic basis behind the emergence/re-emergence of successful clones in outbreaks and measuring the rates at which resistance emerges. In addition, WGS also elucidated some of the etiologic factors that allow pathogenesis and spreading MDR bacteria [93, 143, 190].

WGS revealed that the speed of bacterial MDR evolution depends on the genome plasticity and epidemiology of the pathogen, as well as type and duration of applied antibacterial treatment in healthcare settings. For example, the number of SNPs and structural variations (SVs) was higher in MRSA clones in under-resourced healthcare settings where barriers to transmission were lower [227]. Furthermore, the number of SNP differences between isolates belonging to the same outbreaks positively correlated to the time of their isolation in case of MRSA and *Mycobacterium tuberculosis*, pathogens which are transmitted strictly from human to human within a hospital community [52, 95, 127, 227, 258]. In contrast, studies of *Salmonella enterica* subsp. *enterica* and subsp. *typhimurium*, pathogens which can be transmitted from human to human indirectly through various sources, did not show any impact on the accumulated SNP numbers [141, 178]. Genomic analysis also extended our knowledge about the origin of MDR evolution in bacterial populations demonstrating that evolution is acquired through at least three ways:

i. Transmission of plasmids bearing diverse antimicrobial resistance genes between pathogens/or horizontal gene transfer with the help of mobile genetic elements (MGEs) [12, 37, 179]
ii. Mutations in bacterial drug-related genes and intergenic regions [2, 47, 48, 52, 71, 74, 99, 247]
iii. Differential expression of genes which mediate drug effects [262]

Acquisition of new resistance genes and virulent determinants by horizontal transfer via conjugation, transduction, or transformation usually is associated with pathogen adaptation to new niches or lifestyles and affects the evolution of its genomic content, leading to clinically significant effects. This evolution mostly underlies the success of the MDR emerging strains and may be a major reason of the outbreaks all over the world. Transmissible plasmids and phages frequently bear resistance genes/cassettes conferring bacterial resistance to one or several different antibiotics and facilitate their transfer through different genera. For example, it was revealed that IncA/C plasmids carry extended-spectrum β-lactamases, AmpC β-lactamases, and carbapenemases among broad host range pathogenic Enterobacteriaceae [63, 73, 100, 158, 210, 212]. They are considered the most common reason of hospital MDR of these bacteria for many old and new generations of the β-lactams, including cephalosporins, penicillins, cephamycins, and monobactams [110, 162] (Table 1). Other clinically relevant plasmids include pTW20_1, harboring *qacA* (encoding antiseptic resistance) and *mer* operon (mercury resistance), and pPR9-like carrying *aadD* (aminoglycoside resistance) and *ileS-2* (resistance to mupirocin) genes, are conjugated between MRSA ST 239 isolates [227] and, possibly, can be transmitted between other staphylococcal strains and species [9, 17].

The horizontal gene transfer of chromosomal genes with the help of MGEs is also important in conferring resistance to a wide variety of antibiotics, particularly towards new ones. For instance, recent retrospective studies of *S. aureus* showed that all emergent MRSA populations differed from methicillin-sensitive *S. aureus* (MSSA) not only in plasmid replacement and content but also in such genetic features as small deletion/insertion polymorphisms (DIPs) and presence of MGEs and resistance genes on the chromosome [230, 231, 241]. Further, it was shown that MDR genes are often associated with the MGEs and, with their help, can be transferred to other bacteria between the same or different species [225, 254]. For example, it was shown that the evolution of methicillin resistance in nosocomial and community-acquired MRSA was mostly arisen by acquisition of the staphylococcal cassette chromosome (SCC*mec* type IV cassette) integrated into the chromosome and carrying the *mecA* or *mecC* genes encoding penicillin-binding proteins, which reduced affinity for β-lactam antibiotics [76, 205].

Other recent large-scale studies extended our knowledge about resistance evolution of *S. aureus* CC398 lineage, the most prevalent emerging pathogen with broad host tropism in many European countries [157, 245]. These works shed light on the nature of MDR in CC398 and questioned its origin and the major reasons of its emergence in clinics. All human-specific MSSA and MRSA isolates carried two unique genetic markers: *ϕ7* bacteriophage and *ϕ3* bacteriophage with human-specific immune evasion genes *chp*, *scn*, and *sak* (only in MRSA) [157]. Based on these studies, it was hypothesized that livestock-associated MRSA has diverged from the human-associated MSSA and that it acquired tetracycline and methicillin resistance genes and lost phage-carried human virulence genes [157, 192, 213]. However, further discrete-trait analyses provided for this lineage did not support the hypothesis about its human origin and left the question about evolutionary routes open [245]. This discrepancy may be explained by the lack of unified and standardized computational methods and interpretative algorithms applied for the WGS data analysis.

The WGS data, accumulating for various bacterial species, also showed that specific acquired determinants (mostly, virulence-related genes or islands) can also be the key reasons of the emergence of MDR pathogens causing outbreaks. For instance, it was shown that Panton-Valentine toxin and *sasX* gene, encoding a surface protein, contributed to the outbreaks caused recently by MRSA in the UK and China, respectively [93, 143]. Further, the *mgrB* gene, encoding a transmembrane protein produced upon activation of the PhoPQ signaling system, was found to be associated with colistin resistance in re-emergent *K. pneumonia* causing nosocomial outbreaks worldwide [190].

Antibiotic resistance can also be caused by spontaneous and induced missense mutations within the antibiotic targets or their binding sites, e.g., gyrase subunits A and B, *gyrA* and *gyrB* (targets of quinolones), RNA polymerase subunit B, *rpoB* (target of rifampicin), dihydrofolate reductase, *alr* (rimethoprim), protein biotin ligase, *birA* (Bio-AMS), or membrane proteins (e.g., multidrug efflux protein *norM*) (Table 1) [99]. For example, WGS revealed the mutations in *blaI*, *blaR1*, as well as in the *mecA* regulone (*mecI-mecR1-mecA*) in MRSA [16]. Similarly, it was demonstrated that the major mechanism of MDR in re-emergent *M. tuberculosis* is primarily arisen by point mutations in *rpoB* (S450L), *katG* (P7 frameshift), *gyrB* (T500N), *embB* (D1024N), *rrs* (A514C and A1401G), and *thyA* (P17L) genes [22, 52, 88, 186, 242].

The genomic information together with powerful bioinformatics tools made it possible to distinguish the molecular pathways responsible for MDR-caused diversity. For example, Darch and colleagues have demonstrated that distinct recombination events were the dominant driver of phenotypic diversity in extant population of *P. aeruginosa* obtained from a single cystic fibrosis (CF) patient (with a weight of recombination relative to mutation, r/m, rate approaching 10) [41]. Other retrospective studies identified the exact unique genetic SNVs in main virulence-related genetic factors of *P. aeruginosa* associated with epidemic CF infection [81]. The increased resistance of emerging MDR *P. aeruginosa* to antibiotics was explained by SNPs enrichment of the efflux pumps which actively transport the toxic compound out of the bacterial cell to avoid contact with the target site [45, 113]. Similarly, the revealed genome-wide recombination events in chromosomal β-lactamase genes *bla*_ADC_ and *bla*_OXA-51-like_, plasmid-borne resistance genes, as well transposon- and integron-derived modules were also proposed as major drug resistance diversification drivers for epidemic strains of *Acinetobacter baumannii* [215, 254].

Revealed SNPs and SNVs can be potentially used as a molecular clock to prognose new or potentially emerging/re-emerging outbreak strains, precise tracking, early warning, and targeted infection control of pathogenic bacteria. For instance, the time frame for the emergence of a bacterial pathogen clone and its evolution during epidemic spread had been estimated for MRSA [95]. On the basis of the WGS data, the level of nucleotide substitutions was estimated at 1.68 × 10^−6^ substitutions per site per year in the BEAST analysis, or 2.72 mutations per megabase per year in the parsimony [245, 258]. This translates to approximately one mutation per genome every 6–10 weeks [95]. Taking into account that 1–3 point mutations or large genetic rearrangements (recombination more than 100 bp) in targets related with drug resistance are enough to make differences in antibiotic susceptibility, the provisional prediction of an emergence of novel MRSA clones in clinical settings can be afforded [53, 95]. However, another work demonstrated that using a simple threshold of a maximal number of mutations to rule out direct transmission and emergence of MDR *M. tuberculosis* led to inaccurate interpretation of the data [52]. These authors showed that about 38 % of all individual SNPs were involved in resistance of MDR *M. tuberculosis* and made an important contribution to evolution and emergence of MDR in the bacteria within a single patient [52].

In summary, together with developed tools for WGS data analysis (e.g., Rainbow [264]) and unifying genome-wide database (e.g., *M. tuberculosis* Variation Database (GMTV) [29], The Bacterial Isolate Genome Sequence Database (BIGSdb) [116]) containing the broad spectrum information about individual mutations of pathogens, WGS can be a powerful tool for the preliminary prediction of drug resistance, geographical origin, as well clinical strategies and outcomes.

#### Diagnostics and control of MDR bacterial infections

**Fig. 1:**
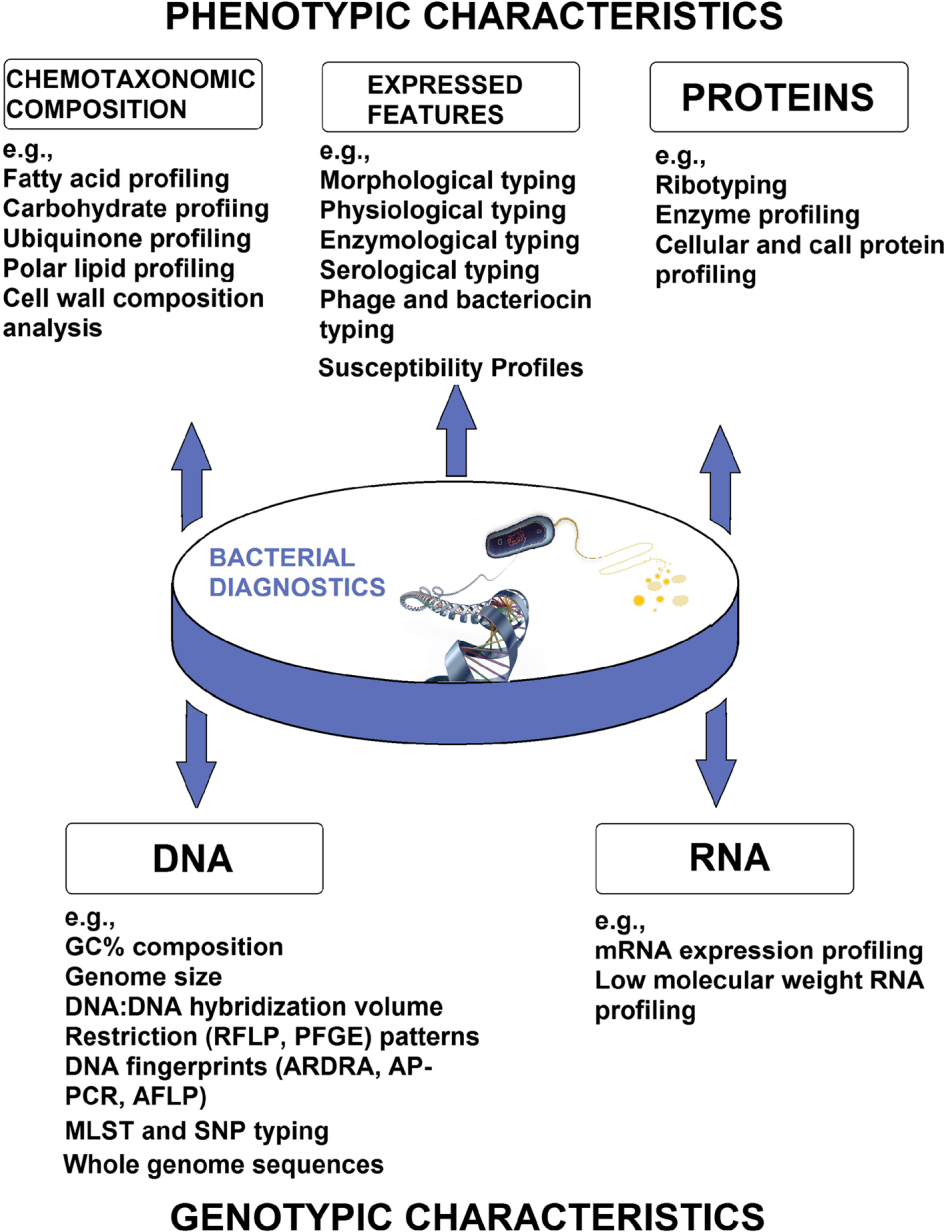
Main characteristics used for the identification and diagnostics of pathogenic bacteria

Successful containment and prevention of MDR infections involves (i) timely identification and characterization of the MDR infectious/outbreak cause, and (ii) discovery of its source and transmission pathways [86, 222, 249]. A significant transformation in MDR infectious disease diagnostics has occurred during the past few decades, including key changes in basic concepts, data analysis approaches, and, especially, methods of exposure measurement and pathogen surveillance [10]. Today, diagnosis of DR pathogenic bacteria are mainly done by means of expensive and time-consuming experimental approaches, including complex phenotypic and genotypic standardized methods [68, 169, 205, 206, 222, 235] (Fig. 1). The techniques applied for this task are mostly based on the detection of phenotypic and genetic traits related to drug resistance, pathogenicity or survival mechanisms of pathogens. Standardized culture-based methods [235], traditional typing (such as biotyping, antibiograms, resistograms), and molecular typing techniques [68, 206, 222] are widely used to detect and identify the cause and course of the outbreaks in the clinical laboratories. Over the last few years, these methods have improved dramatically: they have incorporated automation to increase speed, discrimination power, and throughput, and reduce cost. However, none of these methods is considered optimal for all forms of research and infections. Choice of the method significantly depends on the epidemiological problem to solve, time constrains, its reliability, accuracy, and geographical scale of its use [206]. Furthermore, almost all available approaches have limitations detecting pathogenic microorganisms with rapid transmission dynamics and mutational rates [169], or mixed MDR infections involving multiple unrelated strains or outbreaks caused by closely related isolates [201]. As a result, existing integrated approaches are laborious, time-consuming, expensive, and can lead to misdiagnosis.

Although most of the WGS investigations were retrospective, they demonstrated that WGS technology may make real-time genomic diagnostics a reality [117]. In contrast to multifaceted algorithms used in standard testing, genomic data can provide rapid and accurate detection and control of emerging MDR pathogenic strains in a single process, reducing unnecessary infection-control measures [228]. The genomic information affords unprecedented and detailed insight into microevolution of pathogenicity factors, antibiotic resistance, and transmission mechanisms of pathogens, and, thus, allows robust detection and control of the spread of closely related pathogenic isolates in the clinics [14, 130, 142, 239], communities [30, 72, 77, 84, 159, 203], and globally [15, 94, 95, 168, 227].

The first application of WGS technology was for MRSA, the leading cause of healthcare-associated infections worldwide [45, 55, 171, 172, 258]. WGS techniques detected closely related MRSA clones associated with putative outbreaks, which could not be confirmed with conventional methods, and allowed the reconstruction of local and intercontinental spread of MRSA lineages [53, 93, 95, 127, 130, 258]. For instance, Harris and colleagues studied a putative MRSA outbreak on a special care baby unit at a National Health Service Foundation Trust in Cambridge, UK. During these studies, the cause of a persistent outbreak, a new type ST2371 with Panton-Valentine leucocidin encoding gene, was revealed. WGS technique provided the best discrimination between closely related bacterial clones of the same MRSA lineage, compared to multilocus sequencing typing (MLST) analysis [93]. Importantly, this study resulted in a fundamental shift in the understanding of transmission dynamics and sources of successful epidemic MRSA clones between healthcare facilities and communities. WGS provided strong evidence that community-associated MRSA can be carried for a long period by healthy people [75, 93] and become the cause of healthcare-acquired MRSA infections replacing dominant healthcare-associated lineages [80]. These data facilitated improved infection-control measures for the infectious sources (e.g., workers, visitors, equipment). Later, this study was complemented with more detailed investigations of cause and sources of hospital- and community-associated MRSA lineages in settings with extensive and poor infection-control practices all over the world [157, 227, 248]. It was shown that low resource countries can be the main source of the global emerging MRSA [227]. Thus, the population of MRSA ST239 lineage, aka the Brazilian clone most prevalent across the globe, was significantly more variable (evolved faster) in countries with low-cost prevention planning and implementation than in those with well-resourced healthcare settings [200, 227]. Another work provided evidence for frequent transfer of most prevalent human- and animal-associated MDR MRSA CC398 lineage and indicated that livestock and animals could be the main source of infection in humans [245]. The fact that *S. aureus* could be transferred between humans, animals, and livestock (probably in all directions) raised the main concern for clinicians. Together with evidence for higher levels of MDR in the livestock-associated clades, this raised the need to change the existing biosecurity control in agricultural settings.

Pallen and colleagues were the first who applied WGS technology to study the prolonged hospital outbreak of MDR *A. baumannii* in Birmingham, England, between July 2011 and February 2013. With the help of WGS, a novel isolate, the causative outbreak agent was revealed [142, 177]. This clone could not be detected by conventional methods. As in the case of MRSA, it was revealed that early transmission events can occur through the ward-based contact and environmental contamination of the hospital environment [177]. This knowledge led to tighter ward decontamination procedures and infection-control interventions with the purpose of reducing the risk of further transmission.

WGS has shown potential for elucidation of the transmission dynamics of the MDR *Salmonella* species [6, 177] and for the detection of various epidemic *S. enterica* subspecies [141, 174, 175]. MDR and highly clonal lineages of *K. pneumonia*, an important opportunistic pathogen associated with nosocomial and community-acquired infections [189], can be also successfully detected through WGS [151]. In addition to results for MRSA and *A. baumannii* which showed strong evidence of transmission via alternative routes (e.g., silent transmission vectors), the retrospective genomic analysis of the nosocomial carbapenem-resistant *K. pneumonia* isolates together with epidemiological data revealed unexpected transmission, perhaps through asymptomatic carriers or inanimate objects (ventilators, equipment). In addition, it was concluded that combination of the genomic and patient trace data together with algorithms which accounted for *K. pneumoniae*’s capacity for silent colonization can be used for more effective control of the outbreaks and reconstruction of the most likely pathogen transmission routes [216].

WGS analysis allowed identification and tracing of MDR *M. tuberculosis* more precisely than the currently used conventional typing methods [67, 77, 121, 152, 202, 209, 242]. Using WGS technology, Walker and colleagues first analyzed TB cases of the community outbreaks in the UK Midlands. Only genomic data allowed elucidation of the genetic diversity and detection of closely related mycobacterial genotypes causing these outbreaks [242].

Due to the complexity of antibiotic susceptibility regulation mechanisms in *P. aeruginosa* and the high level of its diversity, the most indisputable WGS implication was usually related to diagnostic and control of CF infections [41, 165]. A number of recent studies of MDR *P. aeruginosa* from a single patient have shown that this technology has a great potential for routine diagnostics and antibiotic susceptibility detection in a clinically relevant time frame [41, 124, 247]. It was proposed that further investigation of the enabling gene pool and resistance mechanisms of MDR *P. aeruginosa* populations could improve clinical outcomes of antibiotic sensitivity and detection testing in the future [41].

Besides the retrospective studies, the real-time WGS analysis was successfully applied for rapid detection of infections and outbreaks caused by neonatal MRSA [53, 130], verocytotoxin-producing *E. coli* (VTEC) [114, 120], *Legionella* sp. [198], carbapenem-resistant *K. pneumoniae* [216], *C. difficile* [53], and *A. baumannii* [204]. For instance, in 2011, real-time WGS clarified the cause of a very mysterious outbreak in a farm in Germany. The outbreak was caused by enteroaggregative *E.coli* O104:H4 clone, epidemiologically linked to human cases and transmitted via contaminated seeds [84, 195, 203, 233]. Another modification of real-time WGS analysis, direct real-time WGS (sequencing clinical specimens without the need for culture), was successfully applied for identification and characterization of slowly growing and difficult-to-culture pathogens in clinical samples [7, 98, 150, 211]. Whereas direct WGS is considered as not cost-effective and less sensitive for some clinical workflows (e.g., in the case of fecal samples or mixed infections) [126], single-colony sequencing is considered a very promising epidemiological tool which can address multiple clinically relevant questions more accurately and faster in the future [129]. A simple WGS protocol has been developed and tested for the detection of a broad range of pathogenic bacteria (17 most clinically important pathogens) from a single bacterial colony [3, 129]. Once the procedure is validated, this method has a lot of advantages for clinical practice [3]. However, the single-colony WGS method may be difficult to optimize in the case of difficult-to-grow pathogens [41].

Although it is presumed that WGS may become the primary tool to provide pathogen diagnostics and control in clinical and healthcare settings in the nearest future, many obstacles remain [126]. Today, real-time genomic diagnosis is mostly based on the detection of SNP, SNV, and SV of relevant multiple genetic loci selected for typing. The housekeeping, structural, and functional genes and intergenic regions [11, 30, 53, 77, 95, 126, 136, 140, 142, 156, 168, 195, 203, 260], as well as the virulent and resistance factors are considered as clinically important markers and are applicable for benchtop typing [206]. Growing WGS data and advances in sequencing technologies constantly lead to the discovery of new genetic or genomic variations important for bacterial growth, pathogenesis, antibiotic resistance, and survival. However, before being applied for diagnostics, this plethora of biomarkers requires intensive study of their functions and associations with particular phenotypic changes. Subsequently, the simple and unified analytical tools/platforms to readily extract relevant information from the genome and interpret it without complex and computer-intensive analysis should be developed, and the clinical health personnel should have a quick access to them [135, 136, 140, 256]. One example of this strategy is the study of *Neisseria meningitidis* outbreak [57, 78, 115] which occurred at the University of Southampton, UK, in 1997. Jolley and colleagues developed an integrated analysis platform and applied it for the robust interpretation and analysis of WGS data obtained for *N. meningitidis*. As a result, this analysis took only a few minutes and permitted complete resolution of the meningococcal outbreak. While these tools are being developed for self-contained laboratory workflow, the integration of the WGS technology with phenotypic, molecular typing methods [39, 40], new strategies of sample and culture selection [68], and epidemiologic data analysis is already enhancing our ability to control and prevent nosocomial or healthcare-associated infections.

#### Development of new diagnostics markers and assays

While WGS sequencing is highly informative, it is not cheap, fast or readily available for screening DR bacterial isolates in various healthcare settings today. For example, current WGS technologies may be too slow for point-of-care diagnostics. As a result, target-specific PCR, real-time PCR, and related technologies [160, 223] still remain the most common methods used in clinical practice. However, it still remains critical to select specific sequences (signatures/targets) for designing molecular assays for the pathogen of interest [5]. In this case, WGS can act as a precursor to generate specific diagnostic tests for timely case definition [102, 193, 219]. The genomic data should be analyzed using computational methods (e.g., KPATH, TOFI, Insignia, TOPSI, ssGeneFinder, or alignment-free methods) in order to identify pathogen signatures, estimate their evolutionary rates across the group, and design highly specific diagnostic assays for target groups of pathogens [104, 193]. Due to the obtained WGS data, numerous novel diagnostic genetic targets have been suggested for routine diagnostics of several pathogenic bacteria over the last few years. An extensive list of putative markers is presented in Table 2. WGS technology can also provide robust information about the reliability of the existing and implemented diagnostic markers and thus can help in avoiding false-negative and false-positive results. For example, the obtained WGS data improved the current diagnostic, cultural, and molecular tests for several pathogens: *S. aureus* [184], TB [125], *E. coli* [51], and *K. pneumoniae* [48].

**Table 2:** List of the putative genetic markers obtained by WGS for diagnostics of the bacterial agents of epidemiological importance

Potential target	Location	Target identity	Pathogen	Ref.
*mecA*/*mecC*	Chr	Adapter protein/Penicillin-binding protein 2a	MRSA/*S. aereus*	[184]
*tetM*	Chr	Tetracycline resistance protein	Livestock-associated *S. aereus CC398*	[157]
*ϕ3*/*ϕ7*	Chr	Bacteriophages	Human-specific *S. aereus CC398*	[157]
*Chp*		Chemotaxis inhibitory protein		
*Scn*		Staphylococcal complement inhibitor		
*Sak*		Staphylokinase		
gp20	Chr	Putative prophage DNA transfer protein	Verocytotoxin-producing *E. coli O104:H4*	[102, 193]
*impB*	Pl	DNA polymerase type Y		
*usid000007 (contig 69, 14714:14853)*	Chr	Sequence positions 47–69 similar to *Ricinus communis* putative receptor serine-threonine kinase mRNA (XM_002525007.1)		
*usid000002 (contig 43, 1486:1633)*	Chr	Positions 4–34 similar to *Pseudomonas putida* BIRD-1 major facilitator transporter protein coding sequence (ADR60257.1)		
*ISAba1*	Chr	Transposase of ISAba1, IS4 family	MDR *A. baumannii*	[166, 254]
*csuE*	Chr	Chaperone-usher pili assembly system	MDR *A. baumannii*,	[254]
*bla*_OXA-51_				
		Bla_OXA-51-like_ beta-lactamase	GC2 (SG1)	
Coding SNP:	Chr		Colistin-resistant *K. pneumonia* KPNIH1	[216]
ind(GA) 321 in KPNIH1_08595 CTG→ATG		Microcin B17 transporter		
(L→M) at 130 in KPNIH1_18808 ACC→ATC		Putative membrane protein		
(T→I) at 1106 in KPNIH1_07189 GGC→TGC		l-Ala-D/l-Glu epimerase and methyl viologen resistance protein SmvA		
(G → C) at 811 in KPNIH1_05438		Putative transport protein		
*ampC*	Pl/	β-Lactamase	β-Lactam resistant Enterobacteriaceae and *P. aeruginosa*	[110, 162, 247]
*ampD*	Chr	*N*-acetylmuramyl-l-alanine amidase, negative regulator of AmpC,AmpD		
*ampR*		HTH-type transcriptional activator AmpR		
*ampG*		Putative transporter		
*gyrA* (Thr83Ile)	Chr	DNA gyrase subunit A	*P. aeruginosa* resistant to fluoroquinolones	[247]
*parC* (Ser87Leu)		DNA topoisomerase 4 subunit A		
*bla*_*VIM-2*_	Pl	Beta-lactamase class B VIM-2	*P. aeruginosa* resistant to β-lactams except monobactams	[247]
*aacA29a*/*aacA29b* putative aph ant(4′)-IIb	Chr^a^	6′-*N*-aminoglycoside acetyltransferase type I, phosphotransferases	Aminoglycoside resistant *P. aeruginosa*	[247]
*mexAB-oprM*	Chr	Efflux pumps and multidrug resistance operon repressors	MDR *P. aeruginosa*	[247]
*mexCD-oprJ*				
*mexEF-oprN*				
*mexHI-opmD*				
*mexR*		Operons respective regulator Genes		
*nfxB*				
*mexT*				
*mexG*				
*mexMN*				
*mexVW*				
*mexXY*				
*muxABC-opmB*				
*emrAB-opmG*				
SMR		Small multidrug resistance family of proteins		
*triABC*	Chr	Presence triclosan efflux pump operon	Triclosan resistant *P. aeruginosa*	[247]
*mexJKL*		Resistance nodulation cell division efflux pump		
*czcCBA*	Chr	Cobalt-zinc-cadmium efflux resistance operon	Heavy metal resistant *P. aeruginosa*	[247]
*pmrAB*	Chr	Membrane bound sensor	Colistin and polymyxin resistant *P. aeruginosa*, *S. enterica* subsp. Typhimurium, and *A. baumannii*	[139, 247]
*phoPQ*	Chr	Kinases and cytosolic response regulator		
60 SNPs		Intergenic regions, enzymes, regulatory and membrane proteins	*S. enterica* subsp. Enteritidis	[87, 176]
*iniBAC*	Drug efflux operon		*M.tuberculosis* resistant to rifampicin, isoniazid, fluoroquinolone ethambutol, amikacin, para-aminosalicylic acid	[52]
*rpoB* (S450L)				
*katG* (P7 frameshift) *gyrB* (T500N)				
*embB* (D1024N)				
*rrs* (A514C, A1401G) *thyA* (P17L)				

*Ch* chromosome, *Pl* plasmid

^a^Except plasmid location in rifampin-resistant *P. aeruginosa* PU21

#### Developing new antibacterial drugs

**Fig 2:**
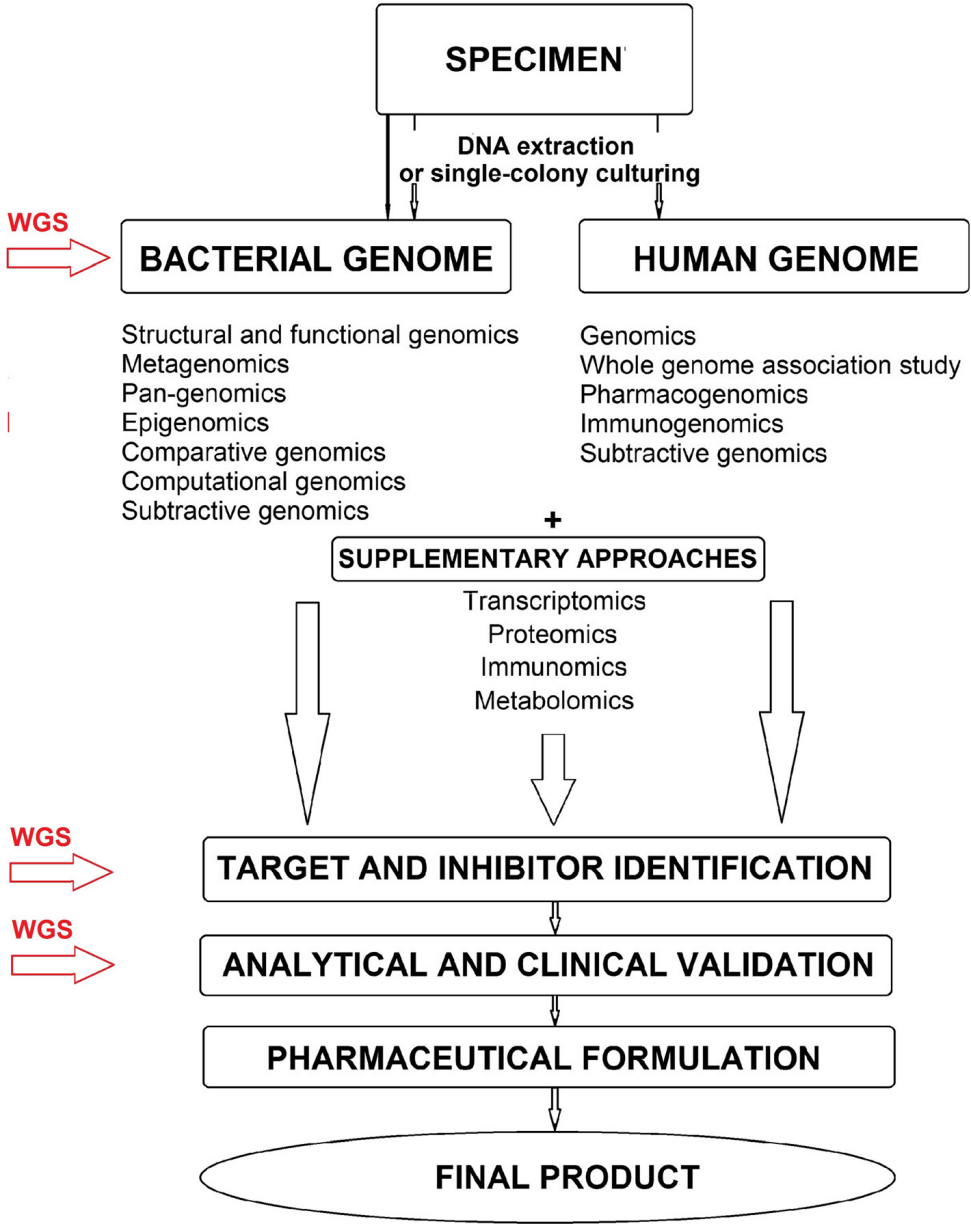
Schematic procedure of drug development based on genomic data, obtained by WGS

Today, a lot of strategies are applied to optimize the identification of new targets and their inhibitors (antibacterial compounds, hits) for the discovery of new antibacterial drugs [50, 214] and predict the mechanisms of their action and their effects in patients. However, clinical management of drug-resistant strains still remains cumbersome. At the same time, the number of newly approved drugs per year has been decreasing, and only five new antibiotics were approved since 2003 [18, 49]. WGS can assist this effort by accelerating the discovery of novel antibacterial inhibitors and targets overlooked by conventional discovery platforms, e.g., sputum smear, culture, and drug susceptibility testing. The innovative WGS technologies can be successfully applied for clinical trials to evaluate the potential antibacterial targets, inhibitors, efficacy of the drugs, and therapeutic alteration of the microbiome in a range of conditions for rational structure-based drug design in a single step (Fig. 2). An important point is that the WGS strategies of screening for novel “drugable” classes of molecules and targets are easily compatible with natural product discovery programs and existing phenotypic high-throughput screening and thus can significantly improve and speed up current practical outcomes [13, 35, 108, 148].

##### Inhibitor-first approach (reverse pharmacology)

The inhibitor-first strategies are more effective than target-driven ones [220] and remain the main approaches of choice for delivering antibacterial drugs to the clinics [20]. WGS screening can be applied to identify molecules that inhibit bacterial growth by diverse mechanisms, including those that engage multiple targets. An extensive list of the antimicrobial drugs discovered recently via WGS data is presented by Deane and Mitchell [44]. As a whole, most of these natural products are essential components of the metabolic pathways for the vitamin biosynthetic (B1, B3, B9), fatty acid synthesis (FASII), and isoprenoid biosynthesis (fosmidomycin, 6-fluoromevalonate). Genomic analysis can also help to reveal genes or gene clusters that are important for biosynthesis of natural antibacterial inhibitors but remain silent under laboratory growth conditions or in the environment. For example, induced expression analysis of environmental DNA gene clusters revealed that tetarimycin A, a new class of tetracylic MRSA-active antibiotic isolated from the culture broth extract of *Streptomyces albus*, was encoded by the *tam* gene cluster [119]. Screening of libraries of complete genomes of the soil microbial community extended the potential value of this compound having revealed numerous silent *tam*-like gene clusters that possibly encode other members of tetarimycin family in the environment [119]. *Streptomyces coelicolor* is another example. Before completion of its genome sequence, only three gene clusters coding natural products had been identified for actinorhodin [154], prodiginine [58], and lipopeptide calcium-dependent antibiotic [32]. WGS revealed that *S. coelicolor* carries clusters of new “cryptic” genes which have a potential for biosynthesis of 29 structurally complex unknown natural products that can be potentially applied as antimicrobials [38].

##### Target-driven approaches

Knowledge of the three-dimensional structure of the drugable targets can also be used for generating or discovering novel-specific inhibitors. Traditionally, a target-driven approach starts from high-throughput screening for inhibitors of a purified target protein. Unfortunately, most inhibitors identified in high-throughput screenings are not active against live bacteria or are not safe for use in humans [185]. WGS can contribute to the de bene esse discovery of the candidate genetic targets for both inhibitors of known or entirely novel mechanism of action (MOAs) before conventional screening for DR bacteria. Determination of resistance mutations in the targets by WGS can also be used for evaluation and estimation of the resistance of the bacterial population to the drug. The target-driven WGS approach was first applied for target FabF, an enzyme required for fatty acid biosynthesis (FAS) [122]. Four novel type II FAS (FASII) inhibitors with broad spectrum activity against Gram-positive bacteria, including MRSA, Platensimcyin, Plantencin, BABX, and Phomallenic acid C, were developed using this method [19, 122, 207, 244, 259]. Recently, several novel antibiotics, fasamycin A and B, with specific activity against FabF of MRSA and vancomycin-resistant *Enterococcus faecalis* were also revealed [61].

Studies performed on a collection of several human pathogens suggested that on the average, about 15–25 % of all genes in a genome are potential drugable targets [33, 164, 238]. These studies concluded that the potential targets are regions whose products/structures are important for bacterial growth and survival under a variety of conditions (e.g., the synthetic machinery of the bacterial membranes, peptidoglycans, lipopolysaccharides, the DNA replication machinery, the nucleic acid synthesis pathway, and ribosomal structures) but do not prevent growth in animals or humans [243]. Thus, WGS screening identified mutations correlating with mycobacterial MDR in genes involved in respiration, fatty acid biosynthesis *kasA* [137], *qcrB* [1, 187], protein synthesis *aspS* [89, 107], protein secretion *eccB3* [107], polyketide biosynthesis *pks13* [107, 246], mycolic acid transport *mmpL3* [197], and arabinogalactan synthesis *dprE1* [34]. Another study of pathogenic bacteria revealed other candidate structures e.g., amino-acyl-tRNA binding site (A-site) and components of the 2-C-methyl-d-erythritol 4-phosphate (MEP) pathway which are also potential targets for the development of new antibiotics for various emerging pathogens [105, 186]. Screening of bacterial genomes for the presence of this ligand can be used for the development of drugs which are active against a wide range of pathogens [64, 105, 236].

However, the target-driven method has some limitations. For example, it can only be applied if resistant strains were obtained. Furthermore, it is important to remember that the target-mechanism identified (such as efflux pump expression, chemical inactivation, or malfunction of transforming an inactive prodrug into the active derivative) can be just one of the existing mechanisms by which mutations can impart resistance. Presence of several candidate targets, which belong to the same protein family with conserved inhibitor binding, can also complicate their further interpretation and evaluation by overexpression analyses [21, 234]. In addition, mutations in nonessential genes can also significantly modulate the main target’s structure or functionality resulting in partial activity of antibiotics [147].

##### Clinical trials

WGS can be applied to design clinical trials more efficiently. First, it can be used at the early phases of drug development to screen a phylogenetically diverse collection of the pathogens for the presence and variability of the candidate drug’s target. Such analysis will prove that this target and its variations are valid and important for all species and lineages of the pathogenic genus and, thus, reduce the chance to miss any resistant strains [128].

Second, WGS can be used to determine drug’s MOA directly. Although it is not mandatory to define an antibacterial compound’s MOA for use in humans, this knowledge can help developing novel drugs for a broad range of bacteria and evaluate their toxicity and specificity a priori. Knowledge of MOAs will also reduce time for clinical trials of chemically redundant putative compounds that fail for the same mechanistic reasons. Further, identification of the MOA and candidate targets can give another chance to existing antibiotics. For example, bottromycins, antibacterial peptides with activity against several Gram-positive bacteria and mycoplasma, were discovered more than 50 years ago. Later on, it was revealed that these peptides’ binding A-site on the 50S ribosome lead to the inhibition of protein synthesis and thus can become a novel promising class of antibiotics applied against vancomycin-resistant Enterococci (VRE) and MRSA [105].

Third, knowledge about resistance mechanisms at the genetic level is very important for determining and avoiding cross-resistance of the pathogen, when multiple antibiotics should be applied for treatment [167]. Fourth, sequencing of pathogens during clinical trials has the potential to distinguish exogenous re-infection from the primary infection. This is crucial in order to assess the efficacy of study drugs and estimate the therapeutic effect in a range of conditions [22, 23, 127, 237].

However, as the field of the genomic drug and target discovery moves forward, the problem stemming from the elucidation of novel unknown classes of gene products remains significant. It is important to remember that no single method is sufficient to define the MOAs of most antibacterial drugs, but a complex approach is required [27]. The detailed genomic analysis of the human pathogens (microbiota), as well as gene expression and drug susceptibility analyses of pathogens, together with powerful bioinformatics tools, can provide new applications to “old” drugs and invigorate the discovery process for novel antibiotics [43, 191]. In this regard, the discovery of the novel anti-TB inhibitors (e.g., bedaquiline, pyridomycin, SQ109, BM212, adamantyl ureas, benzimidazole, BTZ, TCA, and imidazol[1,2-a]pyridine related derivatives) succeeded by a combination of high-throughput screening and WGS analysis of spontaneous resistant mutants for target identification, combined with modern bioinformatics tools [8, 97, 183]. Zomer and colleagues also demonstrated that the combination of high-density transposon mutagenesis, WGS, and integrative genomics has a great potential for reliable identification of potential drug targets in *Streptococcus pneumoniae*, *Haemophilus influenzae*, and *Moraxella catarrhalis* [164]. This complex analysis predicted 249 potential drug targets, 67 of which were targets for 75 FDA-approved antimicrobials and 35 other researched small molecule inhibitors.

## Conclusions

What does the future hold for WGS? Herein, we showed that WGS may be well poised to make a decisive impact on the study and control of MDR in pathogenic bacteria (Table 1) [126]. However, although not reviewed here, studies have shown that WGS can also contribute to the investigation of various pathogenic and beneficial resistant microorganisms: bacteria [70, 155], fungi (*Candida* spp., *Cryptococcus neoformans*, *Pneumocystis* spp., and *Aspergillus* spp.) [208], and viruses (HIV virus, hepatitis B, hepatitis C, influenza, herpes viruses) [144, 255]. Of course, we should not neglect the potential importance of the human genome sequencing and investigation of host–pathogen interaction for patient management and drug development. The combination of the MDR bacterial and human WGS data together with genome-wide association studies and expanding computational capacity offers new power to elucidate host immune traits and genetic factors/variants contributing/altering to susceptibility to MDR bacterial diseases in humans [28]. Such studies have been extensively published [4, 28, 65, 226].

Technical development promises portable, single-molecule, long-read, and user-friendly sequencing platforms, with high functionality and cost-effectiveness. These novel technologies will provide unprecedented opportunities for clinics and public health and may soon change our lifestyle. However, there are still many difficulties to overcome. There is a call for conceptual change of rational sampling strategies, experiment design, and data analysis management. The proper collection, processing, and storage of biological specimens are also critical. The pathway from sequencing the DNA of a specimen to a clinical treatment plan of the patient depends on the integration of each sample’s genomic information with databases that contain known genotype–phenotype correlations and clinical associations obtained from large sample sets. Well curated and regularly updated databases of resistance genotype–phenotype links of MDR pathogens and computational tools to interrogate the ever-increasing information in a robust way are urgently required for MDR pathogen identification and control as well as for novel drug development. These improvements will help to solve many of the critical issues of WGS applicability for both public health and scientific purposes.

## Abbreviations

AGST: antigen gene sequence typing
A-site: amino-acyl-tRNA binding site
BSI: bloodstream infection
CA-UTI: catheter-associated urinary tract infection
CF: cystic fibrosis
CI: confidence interval
DIPs: deletion/insertion polymorphisms
DR: drug resistance (or resistant)
FAS: fatty acid synthesis
HAP: hospital-acquired pneumonia
IGS: individual genome sequencing
MALDI-TOF MS: matrix-assisted laser desorption ionization–time of flight mass spectrometry
MDR: multidrug resistance (or resistant)
MGEs: mobile genetic elements
MIRU-VNTR: mycobacterial interspersed repetitive unit variable number tandem repeat genotyping
MLST: multilocus sequencing typing
MRSA: meticillin-resistant *Staphylococcus aureus*
MSSA: methicillin-sensitive *S. aureus*
NGS: next-generation sequencing
PBP: penicillin-binding protein
SCC: staphylococcal cassette chromosome
SNP: single-nucleotide polymorphism (a single nucleotide aberration which can be found in more than at least 1 % members of bacterial population)
SNV: single-nucleotide variation (a single nucleotide aberration without any limitations of frequency e.g., was not validated for population and can be found in one individual)
SSI: surgical-site infection
SV: structural variations (large genomic variations, including insertions, deletions, inversions, translocations, and duplications)
TB: tuberculosis
UTI: Urinary tract infection
VAP: ventilator-associated pneumonia
WGS: whole-genome sequencing
WSI: wound stream infection
